# Parallel transverse uterine incisions combined with cell salvage minimized bleeding in a patient with pernicious placenta previa and an unexplained decrease in hemoglobin after transfusion of allogeneic red blood cells: A case report

**DOI:** 10.1097/MD.0000000000015434

**Published:** 2019-05-03

**Authors:** Yushan Ma, Yong You, Xiaoqin Jiang, Xuemei Lin, Yan Chen

**Affiliations:** a Department of Anesthesiology; b Department of Gynecology and Obstetrics; c Department of Radiology, West China Second University Hospital, Key Laboratory of Birth Defects and Related Diseases of Women and Children (Sichuan University), Ministry of Education, Chengdu, Sichuan Provence, China.

**Keywords:** cell salvage, cesarean section, parallel transverse uterine incisions, pernicious placenta previa, placenta accrete

## Abstract

**Rationale:**

The incidence of pernicious placenta previa (PPP) and placenta accreta (PA) is increasing in China. Excessive blood loss in these women is an important cause of maternal death and emergency hysterectomy. Performing a traditional cesarean section (CS) in women with PPP is stressful for obstetricians because avoiding cutting the placenta is difficult. As a result, sudden life-threatening bleeding may be encountered. Therefore, there is an urgent need to establish a novel operative method for PPP and PA that is safe for both the mother and neonate, and less stressful for the surgeon.

**Patient concerns:**

We report an extremely rare case of PPP and PA complicated with anemia and an unexplained decrease in the hemoglobin (Hb) levels after transfusion of 3 units of allogeneic red blood cells.

**Diagnoses:**

The patient was diagnosed with unexplained anemia, and hemolysis resulting from donor red blood cell transfusion was suspected preoperatively.

**Interventions:**

To minimize blood loss for safety, a new operative technique, parallel transverse uterine incisions (PTUI) in CS (PTUI CS), was used under general anesthesia in this case. Inhaled volatile sevoflurane was used for uterine relaxation during PTUI. Cell salvage was also used.

**Outcomes:**

PTUI CS combined with cell salvage effectively reduced bleeding and preserved the uterus in our patient. Sevoflurane was effective for uterine relaxation during PTUI CS.

**Lessons:**

If PPP and PA are suspected, placental magnetic resonance imaging is recommended for definitively determining whether a transverse fundal incision can be made. If feasible, we strongly recommend that PTUI CS combined with cell salvage are used to minimize bleeding for high-risk patients with PPP and PA complicated with anemia and an unexplained decrease in Hb levels after transfusion of 3 units of allogeneic red blood cells. Anesthesiologists should be vigilant to maintain uterine relaxation from the time of delivery of the neonate to a second transverse incision in the lower segment of the uterus. This is a key element of successful PTUI CS. Additionally, the use of intraoperative cell salvage is recommended when it can be expected to reduce the likelihood of donor red cell transfusion.

## 1 Introduction

The incidence of pernicious placenta previa (PPP) with placenta accreta (PA), which results from an increasing rate of cesarean section (CS), particularly repeated CS,^[1–3]^ is increasing in China. Excessive blood loss is an important cause of maternal death ^[4]^ and emergency hysterectomy in women with PPP. Treatment of major hemorrhage, in addition to transfusion of red cell mass and managing anemia, is strategy for minimizing blood loss. Performing a traditional CS in women with PPP is stressful for obstetricians^[5,6]^ because avoiding cutting the placenta is difficult. Obstetricians must remove the placenta without direct visualization. As a result, sudden life-threatening bleeding may be encountered ^[7]^ and cesarean hysterectomy may be performed to control intraoperative bleeding. Therefore, there is an urgent need to establish a novel operative method for PPP that minimizes blood loss and is safe for the mother and neonate, and less stressful for the obstetrician.

**Figure 1:**
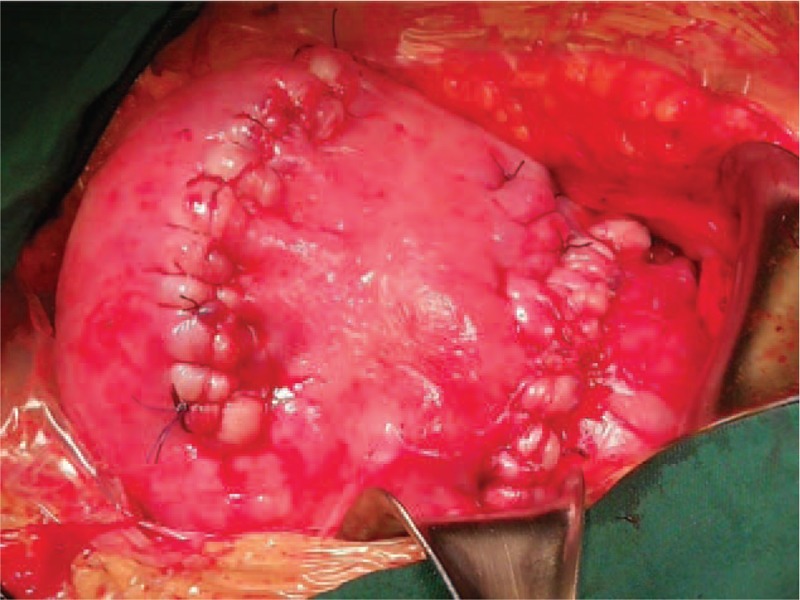
Parallel transverse uterine incisions cesarean section in this case.

We report here an extremely rare case of PPP and PA complicated by an unexplained decrease in hemoglobin (Hb) levels after transfusion of 3 units of allogeneic red blood cells. To minimize blood loss for safety, a new operative technique, parallel transverse uterine incisions (PTUI) in CS (PTUI CS) (Fig. 1) combined with cell salvage was used in this case. This technique effectively reduced bleeding and preserved the uterus.

## 2 Case report

**Figure 2:**
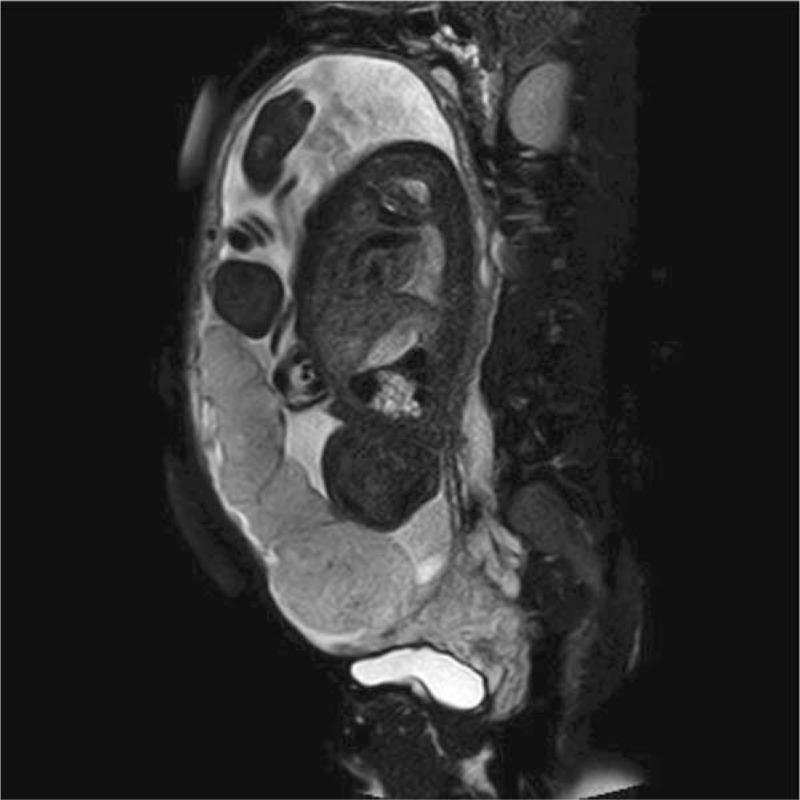
Magnetic resonance imaging (MRI) before cesarean section. It showed the pernicious placenta previa and placenta accreta in the anterior inferior uterine incision scar site.

A healthy 31-year-old woman, G4P3, presented to our hospital during her 4th pregnancy. She had a history of 3 CSs in 2011, 2013, and 2017 in other hospitals. She was 38+3 gestational weeks. Abdominal ultrasonography showed that PPP in the lower edge of the placenta was close to the cervix, and it was suspected to be combined with PA. Placental magnetic resonance imaging (MRI) showed PPP and PA in the anterior inferior uterine incision scar site (Fig. 2). A routine blood examination showed that her Hb level was 72 g/L and her hematocrit was 22.7%. The blood bank supervisor checked the patient's ABO, Rh, and antibody status and found that she had positive antibodies to red blood cells. Because the patient had PPP and PA, life-threatening bleeding could occur during CS. Therefore, 3 units of leukocyte-depleted red blood cell suspension were intravenously infused. This leukocyte-depleted red blood cell suspension did not contain positive antibodies to red blood cells. There was no blood transfusion reaction during this process.

On the 2nd day after allogeneic blood transfusion, the patient did not have any emergency bleeding symptoms, but her Hb level was 68 g/L. Hemolysis was suspected, and thalassemia and hemolysis-related indices were urgently examined. The patient had intermittent established labor. Therefore, the obstetrician decided to terminate the pregnancy immediately. After obtaining consent from the patient, bilateral internal iliac artery (IIA) balloons were placed in her IIA before CS. The patient was then immediately transferred to the operating room. At the time of PTUI CS, the results of hemolysis and thalassemia indices were unknown.

**Figure 3:**
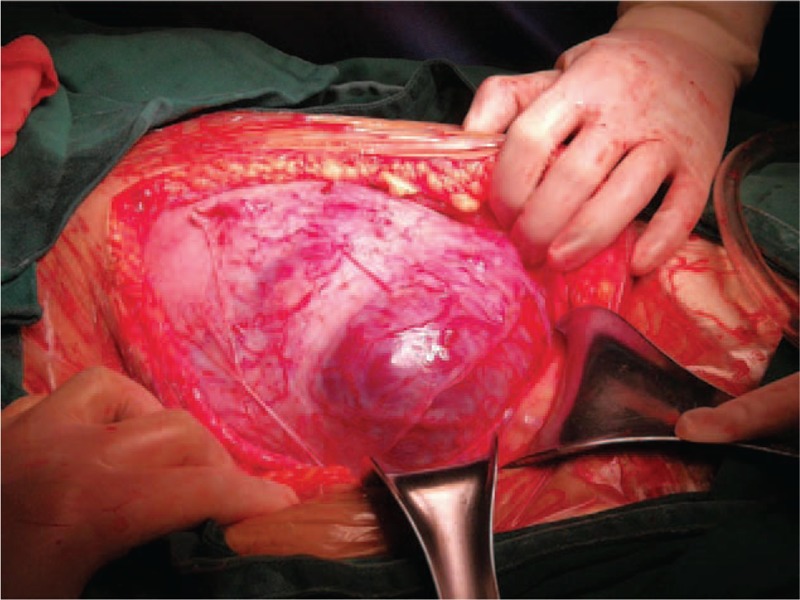
The obvious distension of lower anterior wall and large blood vessels and the placenta penetrating through the anterior uterine wall was observed during laparotomy.

When the patient entered the operating room, blood pressure was 117/74 mm Hg and her heat rate was 84 beats/min. A radial arterial catheter was placed to monitor continuous arterial blood pressure and for measurement of the acid/base status. Her Hb level was 65 g/L. Intraoperative cell salvage, which collects, processes, and returns the woman's own blood lost during surgery, was used. A vertical abdominal incision was made under general anesthesia. Obvious distension of the lower anterior wall, and large blood vessels and the placenta penetrating through the anterior uterine wall were observed during laparotomy (Fig. 3). After confirming the location of the placental margin according to preoperative MRI results, the first transverse incision was made near the uterine fundus and above the upper border of the placenta, without transecting the placenta.

**Figure 4:**
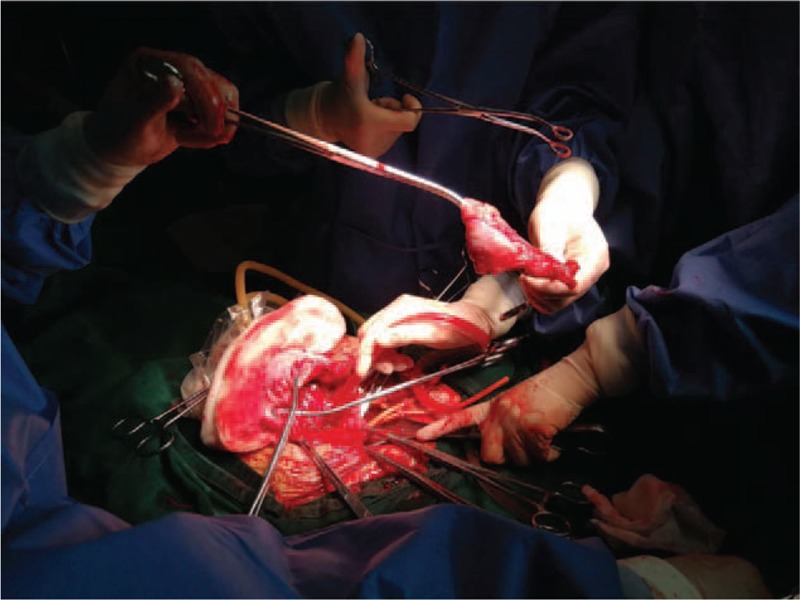
Below the cervix and uterine body under the first fundus incision was ligated tightly by narrow rubber tourniquet, respectively. Wedge resection of the thin uterine wall at the PA site of uterus anterior was performed. Revealed large blood vessels and placenta penetrating through the anterior uterine was observed.

A healthy male infant was delivered from the first incision, and he weighed 2830 with a height of 50 cm. The newborn's Apgar scores were 10 and 10 after 1 and 5 minutes, respectively. The placenta remained in the uterus. Bleeding from the first incision was minimal and was easily controlled by blood vessel ligation. After delivering this neonate, the inhalation concentration of sevoflurane was increased to 2.0 to 3.0 minimum alveolar concentrations to achieve complete uterine relaxation. According to the degree of uterine relaxation, intermittent venous injection of 100 μg nitroglycerin was performed to maintain uterine relaxation. If blood pressure greatly decreased, intravenous injection of 100 μg phenylephrine was performed to raise blood pressure. The first uterine transverse incision was closed by a rapid, continuous, double-layer suture after ligation of the umbilical cord. Bilateral small openings were made in an avascular area of the ligament at the level of the cervix. A narrow rubber tourniquet was then passed through both openings and tied tightly below the cervix to restrict uterine blood flow. The uterine body under the first fundus incision was also tied tightly by another rubber tourniquet to prevent bleeding from this incision. At the same time, prepositioned bilateral occlusion balloons of the IIA were then filled to completely obstruct the blood supply. The inhalation concentration of sevoflurane was decreased to 1.0 minimum alveolar concentration. A second transverse uterine incision was made in the lower segment of the uterus, and the placenta was manually removed under direct observation. Wedge resection of the thin uterine wall at the PA site of the anterior uterus was performed (Fig. 4). The bilateral IIA balloons and the narrow rubber tourniquet for restricting blood flow to the uterine cervix and uterine fundus were then loosened. No bleeding occurred, and the second transverse uterine incision was closed using interrupted sutures. Because the patient already had 3 children, a bilateral tubal ligation operation was performed as strongly requested by the patient. In the whole operation, the total blood loss was estimated to be 1.3 L, and she was transfused 462 mL of recycled red blood cells processed with a leukocyte depletion filter. The lowest recorded Hb level was 54 g/L with a hematocrit of 16%. However, at the end of the operation, her serum hemoglobin level was 63 g/L, hematocrit was 19%, and the activated partial thromboplastin time was normal.

## 3 Discussion

We experienced an extremely rare case of PPP and PA complicated by an unexplained decrease in Hb levels after transfusion of 3 units of allogeneic red blood cells. To minimize blood loss and for the reason of safety, a novel operative technique, PTUI CS, combined with cell salvage was used in this case. This technique effectively reduced bleeding and preserved the uterus.

An important factor in our case was how to choose an effective operation method to minimize bleeding and reduce the possibility of massive transfusion of donor red blood cells. She was diagnosed with unexplained anemia, and hemolysis was suspected from transfusion of blood products before the operation. If massive bleeding occurs during the operation, donor red blood cells are required because of anemia and bleeding. However, an increased use of donor red blood cells may not be safe for suspected hemolysis. At this point, the anesthesiologist and obstetrician have a dilemma. Performing a traditional CS in women with PPP is stressful for obstetricians because of the difficulty in avoiding cutting the placenta to deliver the neonate. This results in massive bleeding. This is not a minor issue, especially when the response time is short. Therefore, there is an urgent need to establish a novel operative method for PPP and PA that is safe for the mother and neonate, and less stressful for the surgeon.

We believe that our findings on use of a new operation technique, PTUI, to minimize bleeding during CS are important. To help understand and apply this new technique, a few points need to be emphasized.

First, placental MRI is effective for assessing the state and location of the placenta. The major benefits of MRI are avoiding trying to detach the placenta during delivery, which inevitably leads to major bleeding and emergency hysterectomy. If PPP and PA are suspected, placental MRI is recommended for definitively determining whether a transverse fundal incision can be made. If feasible, the first transverse fundal incision is performed near the uterine fundus, through which accidentally cutting the placenta can be completely avoided, thus decreasing the risk of massive bleeding. Delivery of the neonate from the first incision is simple and smooth. The uterine incision can then be sutured immediately.

Second, in our case, bilateral small openings were made in an avascular area of the ligament at the level of the cervix. A narrow, red, rubber tourniquet was then passed through both openings and ligated tightly below the cervix to restrict uterine blood flow. The uterine body under the first fundus incision was also ligated tightly by another rubber tourniquet to prevent bleeding from this incision. Prepositioned bilateral occlusion balloons of the IIA should be used if feasible. All of these measures are important, because if bleeding is not controlled, the process may be aggravated and result in coagulopathy or other severe complications. A 2nd transverse uterine incision was then made in the lower segment of the uterus, and the placenta was manually removed under direct observation. The bilateral IIA balloons and the narrow rubber tourniquet for restricting blood flow to the uterine cervix and uterine fundus were loosened. No bleeding occurred, and the second transverse uterine incision was closed using interrupted sutures. This is an important procedure for minimizing bleeding that below the cervix and uterine body under the first fundus incision was ligated tightly, respectively, as shown in our case.

Third, the key element to success of the new PTUI procedure is to sufficiently maintain uterine relaxation. This enables placental perfusion and prevents placental separation during the time from delivery of the neonate to the 2nd transverse incision in the lower segment of the uterus. Preventing premature placental separation is one of the main measures for reducing massive bleeding in PTUI CS. Use of inhaled volatile anesthetics, such as isoflurane or sevoflurane, is recommended to achieve uterine relaxation, and general anesthesia is often performed for this purpose. In our case, we also used sevoflurane. After delivering the newborn, the inhalation concentration of sevoflurane was increased to 2.0 to 3.0 minimum alveolar concentrations to achieve complete uterine relaxation. Sevoflurane (2.0–3.0 minimum alveolar concentration) was an effective anesthetic for uterine relaxation in our case. When the 2nd incision in the lower uterus was performed, we needed to reduce the inhalation concentration of sevoflurane.

Additionally, use of intraoperative cell salvage is clinically effective and can be life-saving, particularly in our case. In the whole operation process, total blood loss was estimated to be 1.3 L, and the patient was transfused with 462 mL of recycled red blood cells processed with a leukocyte depletion filter. Performing PTUI CS combined with cell salvage minimized bleeding and reduced allogenic blood transfusion. At the end of the operation, her serum Hb level was 63 g/L, which was similar to her preoperative Hb level.

Our novel technique, PTUI CS, has some disadvantages. The major concern of this technique is that the risk of uterine rupture during subsequent pregnancy is unknown. In women desiring a future pregnancy, this technique should be performed only when other traditional operative procedures might be dangerous to perform. Patients planning pregnancy in the future must be explicitly advised of the potential risk of uterine rupture. In our patient who had 3 children, bilateral tubal ligation was performed to prevent any future fertility after obtaining consent from the patient. This novel technique also results in a larger wound in the abdominal wall than is necessary for traditional CS. However, we believe that these concerns are relatively minor because of the increase in safety of our technique for mothers and newborns.

Preterm cesarean hysterectomy may be an effective way of controlling bleeding and reducing allogeneic blood transfusion for patients with PPP and PA if the patient does not need to be fertile. Although our patient already had 3 children, she desired to preserve her uterus. Therefore, preterm cesarean hysterectomy was abandoned.

Another important factor in our patient is the reason why the Hb level did not rise, but decreased from 72 g/L to 65 g/L after transfusion of 3 units of allogeneic red blood cells. Unexplained anemia was diagnosed. The patient had no symptoms of bleeding. Except for donor red blood cell transfusion, no fluid was infused before the operation, and there was no possibility of a diluted decrease in Hb level. Because of the history of antibody positivity to red blood cells, and delayed hemolysis result from transfusion of donor red blood cells was suspected before the operation. Thalassemia and hemolysis-related indices were urgently examined. At the time of PTUI CS, the results of hemolysis and thalassemia indices were unknown. However, indices of hemolysis were normal on the second day after the operation, and then the diagnosis of hemolysis was ruled out. At the time of discharge, the reason for the decrease in Hb level from 72 g/L to 65 g/L after transfusion of 3 units of allogeneic red blood cells was unclear.

## 4 Conclusions

If PPP and PA are suspected, placental MRI is recommended for definitively determining whether a transverse fundal incision can be made. If feasible, we strongly recommend that PTUI CS is used to minimize bleeding for high-risk patients with no fertility requirements. Anesthesiologists should be vigilant in sufficiently maintaining uterine relaxation from the time of delivery of the neonate to that of the 2nd transverse incision in the lower segment of the uterus. This is a key element for successful PTUI CS. Additionally, use of intraoperative cell salvage is recommended when it can be expected to reduce the likelihood of donor red cell transfusion. Regardless of the cause of decreased Hb levels after transfusion of 3 units of allogeneic red blood cells, PTUI CS combined with cell salvage was lifesaving for a patient with PPP and PA.

## Author contributions

**Investigation:** Xuemei Lin.

**Resources:** Yan Chen.

**Supervision:** Xiaoqin Jiang.

**Writing – original draft:** Yushan Ma.

**Writing – review & editing:** Yong You.

## References

[1] Wu S Kocherginsky M Hibbard JU Abnormal placentation: twenty-year analysis. Am J Obstet Gynecol 2005;192:1458–61. 15902137 10.1016/j.ajog.2004.12.074

[2] Silver RM Landon MB Rouse DJ Maternal morbidity associated with multiple repeat cesarean deliveries. Obstet Gynecol 2006;107:1226–32. 16738145 10.1097/01.AOG.0000219750.79480.84

[3] Özcan S Karayalçin R Kanat Pektas M Multiple repeat cesarean delivery is associated with increased maternal morbidity irrespective of placenta accreata. Eur Rev Med Pharmacol Sci 2015;19:1959–63. 26125254

[4] Pavord S Maybury H How I treat postpartum hemorrhage. Blood 2015;125:2759–70. 25769619 10.1182/blood-2014-10-512608

[5] Skupski DW Brady D Lowenwirt IP Improvement in outcomes of major obstetric hemorrhage through systematic change. Obstet Gynecol 2017;130:770–7. 10. 28885411 10.1097/AOG.0000000000002207

[6] Shamshirsaz AA Fox KA Erfani H Multidisciplinary team learning in the management of the morbidly adherent placenta: outcome improvements over time. Am J Obstet Gynecol 2017;216:612.e1–5. 28213059 10.1016/j.ajog.2017.02.016

[7] Chatterjee DJ Bukunola B Samuels TL Resuscitation in massive obstetric haemorrhage using an intraosseous needle. Anaesthesia 2011;66:306–10. 21401545 10.1111/j.1365-2044.2011.06629.x

